# Discovery Sulfoglycomics and Identification of the Characteristic Fragment Ions for High-Sensitivity Precise Mapping of Adult Zebrafish Brain–Specific Glycotopes

**DOI:** 10.3389/fmolb.2021.771447

**Published:** 2021-12-20

**Authors:** Huan-Chuan Tseng, Cheng-Te Hsiao, Nao Yamakawa, Yann Guérardel, Kay-Hooi Khoo

**Affiliations:** ^1^ Institute of Biological Chemistry, Academia Sinica, Taipei, Taiwan; ^2^ Institute of Biochemical Sciences, National Taiwan University, Taipei, Taiwan; ^3^ Université de Lille, CNRS, INSERM, CHU Lille, Institut Pasteur de Lille, US 41-UMS 2014-PLBS, Lille, France; ^4^ Université de Lille, CNRS, UMR 8576—UGSF - Unité de Glycobiologie Structurale et Fonctionnelle, Lille, France; ^5^ Institute for Glyco-core Research (iGCORE), Gifu University, Gifu, Japan

**Keywords:** glycomics, glycotopes, sulfoglycomics, mass spectrometry, zebrafish, HNK-1 epitope

## Abstract

Mass spectrometry–based high-sensitivity mapping of terminal glycotopes relies on diagnostic MS^2^ and/or MS^3^ ions that can differentiate linkage and define the location of substituents including sulfates. Unambiguous identification of adult zebrafish glycotopes is particularly challenging due to the presence of extra β4-galactosylation on the basic building block of Galβ1-4GlcNAc that can be fucosylated and variably sialylated by N-acetyl, N-glycolyl, or deaminated neuraminic acids. Building on previous groundwork that have identified various organ-specific N- and O-glycans of adult zebrafish, we show here that all the major glycotopes of interest can be readily mapped by direct nano-LC-MS/MS analysis of permethylated glycans. Homing in on the brain-, intestine-, and ovary-derived samples, organ-specific glycomic reference maps based on overlaid extracted ion chromatograms of resolved glycan species, and composite charts of summed intensities of diagnostic MS^2^ ions representing the distribution and relative abundance of each of the glycotopes and sialic acid variants were established. Moreover, switching to negative mode analysis of sample fractions enriched in negatively charged glycans, we show, for the first time, that a full range of sulfated glycotopes is expressed in adult zebrafish. In particular, 3-O-sulfation of terminal Gal was commonly found, whereas terminal sulfated HexNAc as in GalNAcβ1-4GlcNAc (LacdiNAc), and 3-O-sulfated hexuronic acid as in HNK-1 epitope (SO_3_-3GlcAβ1-3Galβ1-4GlcNAc) were identified only in the brain and not in the intestine or ovaries analyzed in parallel. Other characteristic structural features of sulfated O- and N-glycans along with their diagnostic ions detected in this discovery mode sulfoglycomic work collectively expand our adult zebrafish glycome atlas, which can now allow for a more complete navigation and probing of the underlying sulfotransferases and glycosyltransferases, in search of the functional relevance of zebrafish-specific glycotopes. Of particular importance is the knowledge of glycomic features distinct from those of humans when using adult zebrafish as an alternative vertebrate model, rather than mouse, for brain-related glyco-neurobiology studies.

## Introduction

Glycomics is increasingly geared toward higher sensitivity and throughput, to be more in tune with advances in other omics undertakings. Unfortunately, such emphasis often compromises structural details as individual glycans are now seldom painstakingly isolated for rigorous characterization using a combination of analytical techniques. This is not supported by the fact that most of the novel and interesting structures are more often not expressed at low abundance amid the more common house-keeping glycomic constituents. Arguably, porous graphitized carbon (PGC)–based LC-MS/MS analysis in negative mode of underivatized glycans would provide many structural insights based on both MS^2^ fragment ions and LC retention time (Jensen et al., 2012; Jin et al., 2017), which can resolve some but not all of the structural isomers. MALDI-MS and MS/MS of native, with and without reducing end tag, and sodiated permethylated glycans in negative and positive ion modes, respectively, is another popular approach that has been the primary technique in identifying many unusual structures, especially those from lower animals (Yamakawa et al., 2018; Paschinger and Wilson 2020; Vanbeselaere et al., 2020). Both would afford linkage-specific cleavages critical to discriminate isomeric structures but still suffer insufficient sensitivity, particularly by MALDI MS and MS/MS, which has to be manually performed and be selective, rather than comprehensive.

More recently, we have introduced an LC-MS/MS–based analysis of permethylated glycans in acidic nanoflow conditions that capitalizes on facile formation of diagnostic oxonium ions from protonated species to allow for mapping and relative quantification of all terminal glycotopes (Hsiao et al., 2017). Where necessary, targeted MS^3^ can be tagged on to key oxonium ions to induce elimination of substituents from position 3 that is sufficient to resolve most of the important glycotopes that are based on either type 1 or 2 Galβ1-3/4GlcNAc units, particularly the various fucosylated glycotopes. Our previous successes include differentiating Le^X^ (Delannoy et al., 2020; Lu et al., 2020) and terminal NeuAc-NeuAc- from another common disialylated glycotopes, NeuAc-Gal-(NeuAc)GlcNAc, as found in the brain (Hsiao et al., 2017; Lin et al., 2019). By further separating and hence enriching any sulfated glycans from non-sulfated ones after permethylation (Yu et al., 2009; Cheng et al., 2013), our workflow also enables a very efficient and high-sensitivity sulfoglycomic analysis by negative mode LC-MS/MS (Cheng et al., 2015), whereby the MS^2^ ions at low-mass ends are sufficient to localize sulfates on either Gal or GlcNAc of a majority of sulfated glycotopes commonly found in mammals. It is anticipated, but yet to be proven, that this workflow should be equally effective against more complicated glycotopes from non-mammals that are often decorated with extra Fuc, Gal, GlcA, and other substituents at unexpected linkage positions. The glycome of adult zebrafish exemplifies one such technical challenge.

Using two seminal studies, one on the embryos (Guerardel et al., 2006) and the other on the adult organs (Yamakawa et al., 2018), we now know that zebrafish makes a range of unusual terminal glycotopes including Le^X^ and sialyl Le^X^ that are additionally β4-galactosylated. The challenge is compounded by the presence of all three sialic acids, NeuAc, NeuGc, and KDN (2-keto-3-deoxy-d-glycero-d-galacto-nononic acid), whereby the mass difference between *N*-glycolyl (Gc) and *N*-acetyl (Ac) is equivalent to that between Hex and deoxyHex, and that of deaminated KDN and NeuAc is equal to the mass difference between Hex and HexNAc. Mere MS1 data thus cannot resolve a plethora of organ-specific glycans. Our previous work has relied on extensive MALDI-MS/MS manually performed on each of the major structures, complemented by GC-MS linkage analysis and even NMR and exoglycosidase digestions. The overall picture gleaned is intriguing as, for example, there are brain-, intestine-, and ovary-specific glycan species, as well as those commonly found in all organs examined. At present, no information on sulfated glycans is available, although it is predicted to be present not only by virtue of implicated sulfotransferases in the zebrafish genome data but also by the expression of an HNK-1 epitope containing a terminal 3-O-sulfated GlcA (Metcalfe et al., 1990; Becker et al., 2001; Ma et al., 2017).

Zebrafish is an excellent model organism that is genetically friendly to allow for a whole range of developmental glycobiology studies (Flanagan-Steet and Steet 2013; de Abreu et al., 2020). With each target glycan manipulation, or changes in the expression of any one or several glycosyltransferases, we require not only efficient functional readout but also rapid mapping of anticipated glycomic changes at desirable spatiotemporal resolution, using as little sample material as possible without compromising the requisite structural details. In that respect and in the absence of glycotope-specific monoclonal antibodies other than that against HNK-1 epitopes, it is imperative to develop a high-throughput, high-precision target mapping assay based on a few critical MS^2^ ions that can discriminate the aforementioned isomeric glycotopes. Moreover, no glycomic map is ever complete without accounting for sulfate modification. We intend to explore sulfoglycomes, in a non-biased discovery mode, to determine if zebrafish likewise makes a range of sulfated glycotopes known to be present in mammals, and which are implicated in myriad recognition events mediated by endogenous glycan-binding proteins (Kannagi et al., 2011; Bull et al., 2021). Above all, despite many ascribed functions (Morise et al., 2017; Sytnyk et al., 2020), the glycan carrier(s) for the HNK-1 epitopes remain(s) undetermined due to technical limitations.

In this work, we have selected three organs from adult zebrafish that comprise most distinctive organ-specific differences. We applied our glycotope-centric LC-MS/MS analysis (Hsiao et al., 2017; Khoo 2021) not only to evaluate its efficiency in mapping all the previously discovered glycotopes but also to uncover any sulfated N-/O-glycans or glycotopes, if present. We demonstrate that each of the unique zebrafish-specific glycotopes can be defined by characteristic MS^2^ fragmentation and, where necessary, further verified by MS^3^. We also determined, for the first time, the presence of sulfated glycotopes primarily of sulfated LacdiNAc and 3-O-sulfated Gal and also the sulfated HNK-1 epitope specifically on the brain N-glycans.

## Materials and Methods

### Zebrafish Samples

The source of zebrafish and the dissection of organs to produce the starting materials for brain, intestine, and ovary glycomic analyses are the same as described previously (Yamakawa et al., 2018) but using 5, instead of 20, adult (6–9 months) zebrafish. Dissected organs were pooled for protein extraction, trypsin/chymotrypsin digestions, N-glycan released by PNGase F, and subsequent O-glycan released by reductive elimination, exactly as performed in a previous study (Yamakawa et al., 2018).

### Glycan Derivatization and Fractionation

Glycans in screw-capped glass tubes were permethylated by adding finely grounded NaOH pellets in 200 μl of dimethyl sulfoxide and 100 μl of iodomethane and incubated in a shaker at 4°C for 3 hours. The reaction was terminated by adding 20% acetic acid on ice. Excess iodomethane collected at the bottom of the tube was evaporated off by applying a gentle stream of nitrogen gas, and the neutralized reaction mixtures were then loaded onto a Waters^®^ OASIS MAX cartridge, which was preconditioned with 3 ml of 100% acetonitrile and 3 ml of 100 mM ammonium acetate buffer. After stepwise washing with 3 ml of ammonium acetate buffer and 9 ml of ddH_2_O, the permethylated glycans were eluted successively by 6 ml of 95% acetonitrile (for neutral glycans), 6 ml of 1 mM ammonium acetate in 80% acetonitrile (single negatively charged), and 6 ml of 100 mM ammonium acetate in 60% acetonitrile and 20% methanol (double and multiple negatively charged). Each glycan fraction was further subjected to additional cleanup by C18 Ziptip^®^ prior to MS analysis to reduce contaminants.

### MS Data Acquisition and Processing

Each cleanup permethylated glycan sample was initially screened by MALDI-MS and MS/MS, where needed and signal intensity allowed. Sample aliquots were mixed 1:1 with matrix (2,5-dihydroxybenzoic acid for positive mode, and 3,4-diaminobenzophenone for sulfated glycans in negative mode, 10 mg/ml) in 50% acetonitrile and spotted onto the MALDI plate for data acquisition on an AB SCIEX MALDI TOF/TOF 5800 system. Instrument settings were laser intensity 4,500, 20 subspectra × 250 shots/spectrum for reflector positive mode MS1; laser intensity 5,000, 40 subspectra x 125 shots/spectrum for reflector negative mode MS1, or laser intensity 5,000, 25 subspectra × 100 shots/spectrum for reflector negative mode MS/MS at 1.5 kv CID energy.

For nano-LC-nanoESI-MS analysis, glycan samples were dissolved in 10% acetonitrile, applied *via* an autosampler to an Ultimate™ 3000 RSLC system connected to an Orbitrap Fusion™ Tribrid™ Mass Spectrometer (ThermoFisher Scientific) *via* a PicoView nanosprayer (New Objective, Woburn, MA), and separated with a constant flow rate of 500 nl/min at 50°C on a ReproSil-Pur 120 C18-AQ column (120 Å, 1.9 µm, 75 μm × 200 mm, Dr. Maisch). The solvent system used was buffer A (100% H_2_O with 0.1% formic acid) and buffer B (100% acetonitrile with 0.1% formic acid), with a 60-minute linear gradient of 30–80% B for N-glycans and monosulfated O-glycans, or 25–60% B for non-sulfated O-glycans. The Orbitrap Fusion Tribrid instrument settings were as described previously (Hsiao et al., 2017) using primarily an HCD-MS^2^ product–dependent MS^3^ data-dependent acquisition method, with full MS and HCD MS^2^ (stepped collision energy at 15 ± 5% for positive mode; 50 ± 10% for monosulfated O-glycans and 50 ± 5% for sulfated N-glycans in negative mode) acquired in the Orbitrap at 120,000 and 30,000 resolution, respectively, and CID MS^3^ (30% normalized collision energy) in the ion trap.

All data were manually examined using Thermo Xcalibur v3.0 software and additionally processed by an in-house LC-MS^2^/MS^3^ glycomic data mining tool, Glypick, as described in Hsiao et al. (2017). The intensities of MS^2^ ions representing diagnostic fragment ions of specific terminal glycotopes that passed the preset 5 ppm filtering criterion were summed and outputted as an indicator for glycotope abundance in an Excel format that were used for plotting bar charts. The *m/z* values of precursors producing MS^2^ spectra that contained at least 2 of the targeted MS^2^ ions were further filtered out, mass fitted to N- and O-glycan glycosyl compositions, and successfully assigned output in an Excel format for manual verification.

## Results and Discussion

Our previous study (Yamakawa et al., 2018) has established the major structural characteristics of the adult zebrafish non-sulfated N- and O-glycans, relying mostly on MALDI-MS and MS/MS analysis on each of the assigned glycan peaks. This is rather simple and effort-consuming. To establish a higher throughput analytical platform based on this groundwork, the permethylated glycans were subjected instead to nano-LC-HCD-MS^2^ product–dependent (pd)-MS^3^ analyses (Hsiao et al., 2017) after an initial MALDI-MS screen to ensure decent recovery from using much less starting material of pooled zebrafish brains, intestines, and ovaries. The premises and anticipated advantages are higher sensitivity and coverage relying on as comprehensive as possible automated MS^2^-pd-MS^3^ data-dependent acquisition and subsequent extraction and quantification of the diagnostic MS^2^ and MS^3^ ion intensities attributable to particular glycotopes. Not all MS^2^ spectra are assignable, particularly those contributed by precursors of very low intensities and/or with undermethylation, different adducts, or even unknown modifications arising from sample work-up. Nonetheless, any of the targeted glycotopes carried on these would still be accounted for as long as it produces a decent diagnostic MS^2^ ion.

**FIGURE 1:**
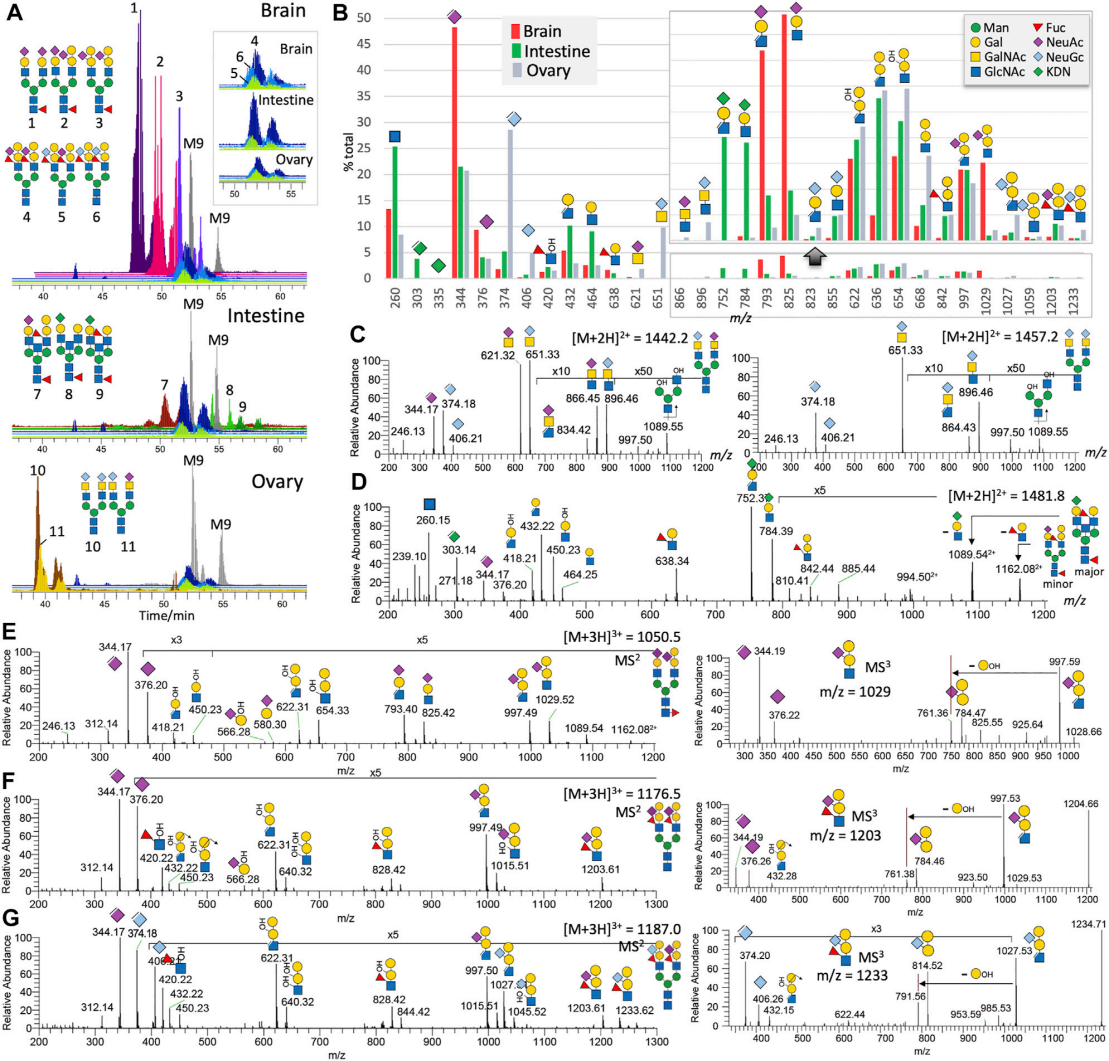
Non-sulfated N-glycans of adult zebrafish. LC-MS/MS analysis of the non-sulfated N-glycans identified at least 11 distinct major complex-type structures, some of which are essentially the same structures but sialylated by different NeuAc and NeuGc combinations. Their brain-, intestine-, and ovary-specific distribution patterns and relative abundances can be inferred from the overlaid XIC plots normalized to those of the commonly found Man_9_GlcNAc_2_ (M9) structure in each of the three organs **(A)**. The overlaid XIC plots for the 3 related N-glycans commonly found in all three organs. The summed intensity for each of the detected diagnostic MS^2^ ions normalized to their total provides an overall assessment of the relative abundance and organ-specific distribution of the three sialic acids and various glycotopes **(B)**. A portion of the bar chart was magnified to highlight the fragment ions of the ±Gal-(NeuGc/NeuAc/KDN)Gal-(±Fuc)GlcNAc glycotopes. The origins of most of the annotated diagnostic MS^2^ ions in (B) can be found in representative sets of MS^2^ spectra shown here for the biantennary N-glycans carrying NeuAc/Gc-sialylated LacdiNAc **(C)**, KDN-sialylated LacNAc **(D)**, Gal-(NeuAc)Gal-GlcNAc **(E)**, Gal-(NeuAc)Gal-(Fuc)GlcNAc **(F)**, and Gal-(NeuGc)Gal-(Fuc)GlcNAc **(G)**, along with the MS^3^ spectra for each of the last three glycotopes (E-G, right panels). A full set of MS^2^ spectra for all the 11 major N-glycans are additionally provided as Supplementary Figure S3. Assigned glycotopes and glycans in this and all other figures were annotated using the recommended Symbol Nomenclature for Glycan (Varki et al., 2015), as shown in the top right inset. Elimination of a MeOH moiety or 3-substituent from the sialic acids and HexNAc creates a double bond, as indicated in the cartoon symbol. Assignment of Gal/Man and GlcNAc/GalNAc is based on prior knowledge of the zebrafish glycotopes as determined in a previous study (Yamakawa et al., 2018). Bisecting GlcNAc is not distinguished from a non-extended terminal GlcNAc on additional antenna.

**FIGURE 2:**
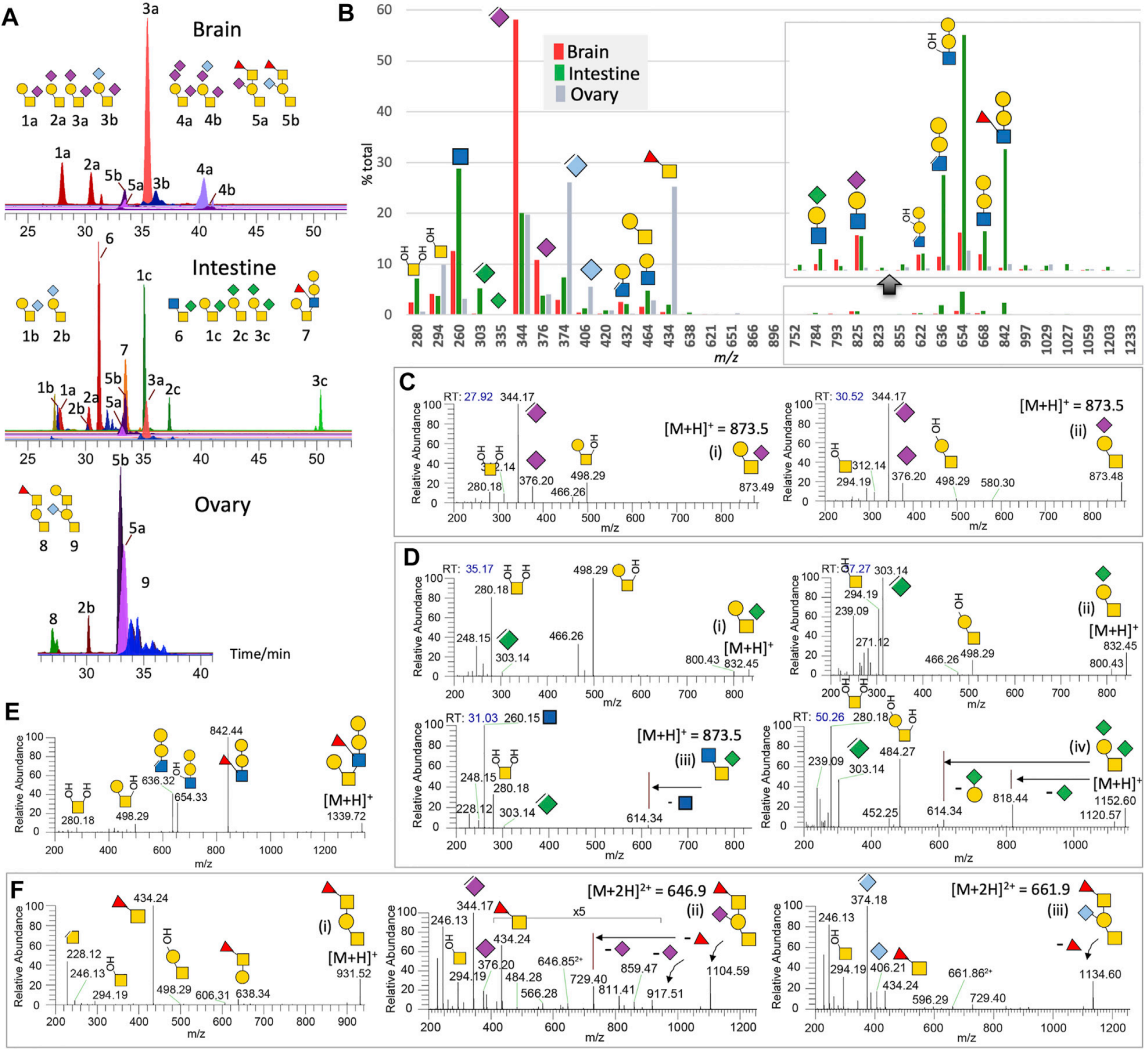
Non-sulfated O-glycans of adult zebrafish. LC-MS/MS analysis of the non-sulfated O-glycans unambiguously identified at least 9 distinct major structures, some of which additionally comprise different NeuAc, NeuGc, or KDN-sialylated versions, with brain-, intestine-, and ovary-specific distribution pattern shown by their respective overlaid XIC plots normalized to the most abundant structure found in each of the three organs **(A)**. Summed intensity for each of the detected diagnostic MS^2^ ions normalized to their total provides an overall assessment of the relative abundance and organ-specific distribution of the three sialic acids and various glycotopes **(B)**. Note that the abundance of KDN is underrepresented by the intensity of its oxonium ions at *m/z* 303/335 relative to those of NeuAc and NeuGc at *m/z* 344/376 and 374/406. *m/z* 335 for KDN^+^ was hardly detectable. A portion of the bar chart was magnified to show only the Gal-Gal-(±Fuc)GlcNAc glycotope defined by ions at *m/z* 842, 668, 654, and 636, and not its additionally sialylated version, and was expressed at a significant level in the intestine alone. The origins of most of the annotated diagnostic MS^2^ ions in (B) can be found in representative sets of MS^2^ spectra shown here for the NeuAc-monosialylated core 1 **(C)**, KDN-sialylated cores 1 and 3 **(D)**, core 2 **(E)**, and core 1 structures carrying the unique Fuc-GalNAc glycotope **(F)**. Assignment of terminal the Fuc-3GalNAc glycotope extending from ±sialylated core 1 structure is based on previous work on the adult zebrafish O-glycans (Yamakawa et al., 2018).

Performed in this way, the analysis is geared toward target mapping of those major glycans and glycotopes previously identified, or based on newly acquired MALDI-MS profiles, as a guide to extract the precursor ion chromatograms (XIC) by accurate mass to identify and quantify major species. This is most effective against the well-resolved, smaller reduced O-glycans but is also applicable to larger non-reduced N-glycans, albeit mostly without resolving their structural isomers. Moreover, each N-glycan would elute as two peaks due to the anomeric separation, which can be collapsed into one if subjected to reduction first prior to permethylation and LC-MS/MS analysis. The final data would be overlapping XICs, showing the relative amount of each identified major species, their respective MS^2^/MS^3^ data, and a tally of all detected MS^2^ ions, as shown in Figures 1, 2 for the non-sulfated N- and O-glycans, respectively, in addition to the initial MALDI-MS profiles (Supplementary Figures S1, S2). A more complete set of HCD MS/MS spectra for the major N-glycans detected are provided in Supplementary Figure S4. Each of the productive MS^2^ scans with precursor ions that can be fitted to sensible glycosyl composition are further listed according to the expected *m/z* values of their corresponding singly charged [M + Na]^+^ and provided in Supplementary Tables S1–S6.

As expected, the MALDI-MS profiles for the permethylated non-sulfated N- and O-glycans from the adult zebrafish brain, intestine, and ovary (Supplementary Figures S1, S2) largely resemble those reported previously (Yamakawa et al., 2018), albeit at a lower signal-to-noise ratio because of using less starting material. Instead of enumerating how many different glycans can be distinguished among all isomeric possibilities over a wide mass range of heterogeneous glycomic constituents, we homed in here on a selected few that are the most characteristic of the three organs (Figures 1A, 2A).

### Non-Sulfated N-Glycans

The ovary N-glycans are distinguished by non-core fucosylated biantennary N-glycans carrying a unique NeuGc/Ac-HexNAc_2_ glycotope corresponding to previously reported α2-6-sialylated LacdiNAc (GalNAcβ1-4GlcNAc) (Hanzawa et al., 2017). Other than the oxonium ion pairs of *m/z* 406 (NeuGc^+^)/374 (ΔNeuGc^+^) and *m/z* 376 (NeuAc^+^)/344 (ΔNeuAc^+^) informing the relative amount of NeuGc vs NeuAc, these unique ovary-specific glycotopes are defined by diagnostic oxonium ions at *m/z* 651/621 and *m/z* 896/866 for NeuGc/Ac-HexNAc^+^ and NeuGc/Ac-HexNAc-HexNAc^+^, respectively (Figures 1B,C). An elimination of the MeOH moiety (−32 u) from the latter indicates that the 3-position of the inner HexNAc is not substituted, which is consistent with it being 4-linked. The intestine N-glycans are characterized by the additional presence of KDN. It is found capping Gal-GlcNAc that is itself not further galactosylated or fucosylated. It could however be paired with NeuAc-Hex-HexNAc or ± Hex-Hex-(±Fuc)HexNAc on core fucosylated biantennary structures. Interestingly, only ΔKDN^+^ at *m/z* 303 and not KDN^+^ at *m/z* 335 was detected. This and the diagnostic oxonium ion for KDN-Hex-HexNAc^+^ at *m/z* 784 and 752 (with a further loss of MeOH) could only be found in the intestine (Figures 1B–D).

The brain N-glycans typically carry a galactosylated Gal-(NeuAc-)Gal-GlcNAc glycotope not found in the other two organs. It is defined by a diagnostic oxonium ion at *m/z* 1,029, accompanied by *m/z* 997 that is indicative of a non–3-substituted GlcNAc. Facile loss of NeuAc from this unique glycotope produced the *m/z* 654/622 pair, whereas further MS^3^ yielded a unique ion at *m/z* 784 corresponding to Hex-(NeuAc)Hex^+^ (Figures 1B–E). Intriguingly, while the additionally β4-galactosylated Le^X^ and sialyl LacNAc were found, respectively, on the intestine and brain core fucosylated N-glycans, β4-galactosylated sialyl Le^X^ glycotope was carried exclusively on non-core fucosylated N-glycans of all adult organs examined (Figure 1A). In fact, it is also the one initially identified in the embryos (Guerardel et al., 2006). This zebrafish-specific glycotope is identified by the diagnostic oxonium ion at *m/z* 1,203 or 1,233, depending on whether it is NeuAc- or NeuGc-sialylated, along with *m/z* 997 or 1,027 upon eliminating the 3-linked Fuc (Figures 1B,F,G). Further loss of the NeuAc/NeuGc generates the common *m/z* 622 ion, while the internal fucosylated HexNAc is further corroborated by the ion at *m/z* 420. This Fuc (HO)HexNAc^+^ ion is complemented by the MS^3^ ions at *m/z* 784/814, corresponding to Hex-(NeuAc/NeuGc)Hex^+^.

Taken as a whole at the glycomics level, the normalized summed intensity of oxonium ions for KDN, NeuAc and NeuGc, and their respective carrier glycotopes (Figure 1B), is fully consistent with previous conclusions (Yamakawa et al., 2018) that brain and ovary N-glycans are mostly but non-exclusively NeuAc and NeuGc-sialylated, respectively. KDN, on the other hand, is found only in the intestine, co-expressed with NeuAc and a very small number of NeuGc. This highly organ-specific presence of KDN in intestine glycoconjugates is believed to originate from the scavenging of microbiota polysaccharide-derived monosaccharides by the intestinal epithelium that uses it as a source of sialic acid to decorate its own N-glycans, O-glycans, and glycosphingolipids at lower energetic expense (Yamakawa et al., 2018). While Gal-Gal-(Fuc)GlcNAc and Gal-(NeuAc)Gal-GlcNAc are characteristics of the intestine and brain, respectively, a fully substituted Gal-(NeuAc/Gc)Gal-(Fuc)GlcNAc is commonly found on all organs and appears to be conjugated to a different class of complex type N-glycans distinguished by lack of core fucosylation. This lack of core Fuc extends to another class of N-glycans carrying NeuGc/Ac-GalNAc-GlcNAc unique to ovary and correlates with a very low expression level of the two FUT8 orthologs fut8A and fut8A in the ovary compared with robust expressions in the brain and intestines (unpublished data).

### Non-Sulfated O-Glycans

The terminal Gal-(±NeuAc/Gc)Gal-(±Fuc)GlcNAc glycotope widely found on N-glycans is not a universal feature of adult zebrafish O-glycans (Figures 2A,B). Among the three chosen organs, only the intestine expresses a non-sialylated version of it on the 6-arm of a core 2 structure, as supported by MS^2^ ions at *m/z* 280 and 498 (Figure 2E). Elimination of the 3-linked Fuc from this glycotope produced the characteristic ion at *m/z* 636 to go along with the ion at *m/z* 842. Other than this structure, the major adult zebrafish O-glycans identified are all based on variably sialylated core 1 structures. The 2 mono-sialylated core 1 structures, 1a and 2a, are well-resolved on C18 LC and can be distinguished by the MS^2^ ion at either *m/z* 280 or 294 (Figure 2C). While those of the brain are predominantly mono- and di-sialylated by NeuAc, and those of the ovary are mostly NeuGc-mono-sialylated, the corresponding core 1 structures are mainly mono- and di-sialylated by KDN in the intestine, a less amount of NeuAc/NeuGc-sialylated versions are also present. Notably, KDN-sialylation shifts the elution time to considerably later when compared to NeuAc/NeuGc-sialylation of the same structure (Figure 2A). As observed for NeuAc-mono-sialylated core 1 structures, both the NeuGc and KDN mono-sialylated counterparts, that is, isomeric structures 1b versus 2b and 1c versus 2c, can be fully resolved on C18 LC (Figure 2A). Unexpectedly, by virtue of this chromatographic separation, the intestine O-glycans at *m/z* 873.5 yielded a third peak that is not a NeuAc-sialylated core 1 but a KDN-sialylated core 3 instead (Figures 2D–2iii). This O-glycan was not previously identified by MALDI-MS/MS (Yamakawa et al., 2018) as the afforded chimeric MS^2^ spectrum containing both NeuAc and KDN, as well as terminal HexNAc oxonium ions, would have confused the assignment.

The dominance of core 1 and 3 structures mono-sialylated at the 6-position of GalNAc by KDN in the intestine is rather unusual. This can be contrasted with the dominance of NeuAc-disialylated core 1 structures in the brain, and the NeuAc/NeuGc-mono-sialylated core 1 structures further extended by the previously identified zebrafish-specific terminal Fucα1-3GalNAc glycotope in the ovaries (Figure 2A). Each of the sialylated and non-sialylated core 1 structures extended by this unique glycotope would afford the diagnostic FucHexNAc^+^ oxonium ion at *m/z* 434 (Figure 2F), which could also be detected in the brain and intestine but only representing very minor O-glycomic constituents there. In summary, the overlaid XICs (Figure 2A) and the summed intensities of diagnostic MS^2^ ions (Figure 2B) indicate that the brain O-glycans are mostly substituted by NeuAc, whereas those of intestines by KDN, Gal-Gal-(Fuc)GlcNAc, and terminal GlcNAc as found in the unique KDN-sialylated core 3 structure. The ovarian O-glycans, on the other hand, are dominated by the NeuGc-sialylation and the unique Fuc-GalNAc glycotope also found in the embryos.

### Sulfated N-Glycans

**FIGURE 3:**
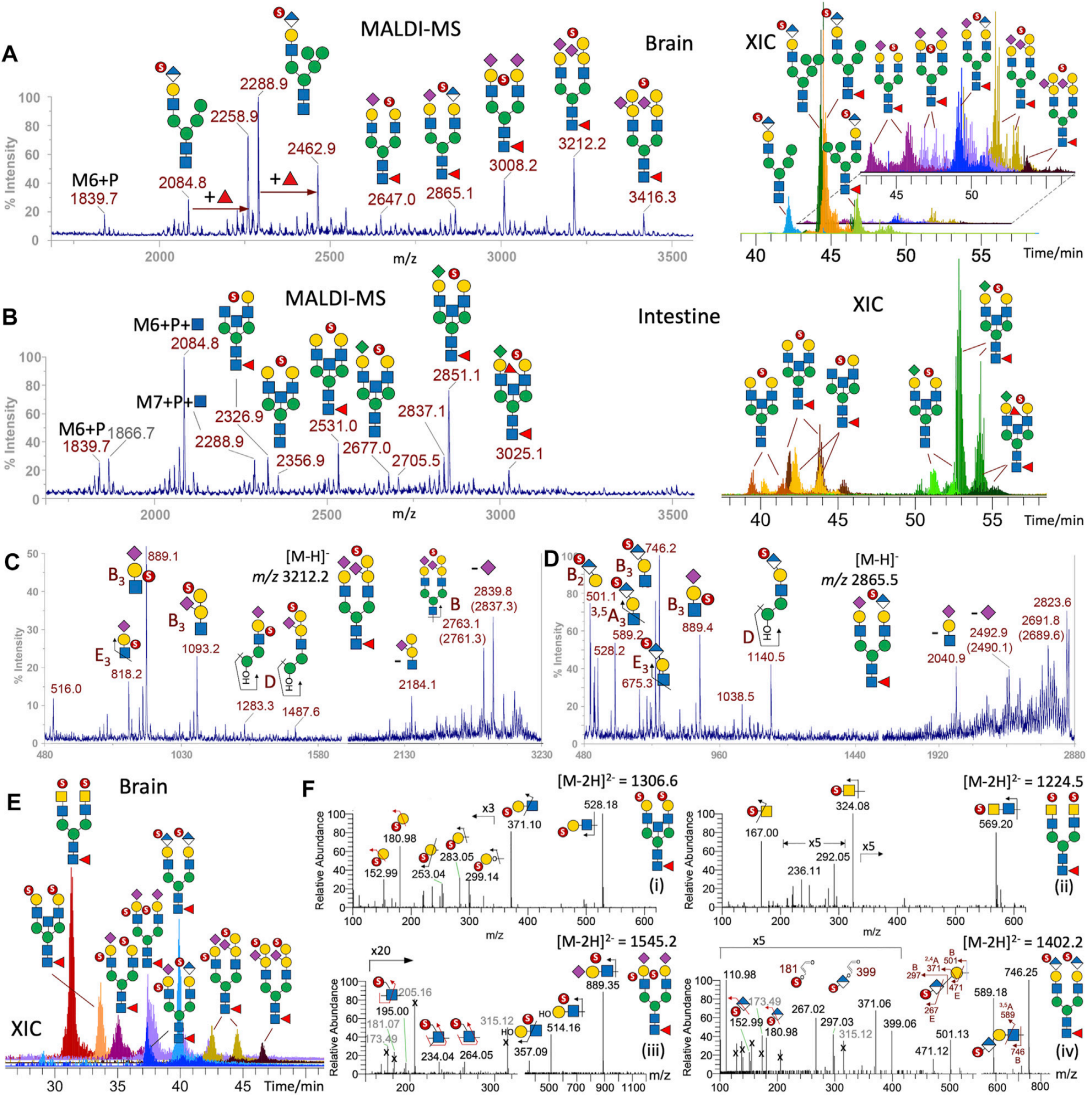
Sulfated N-glycans of adult zebrafish. MALDI-MS screening revealed the presence of permethylated monosulfated N-glycans in the enriched sample fractions derived from brain **(A)** and intestine **(B)** but not ovaries. XICs of each of the major monosulfated N-glycans were overlaid to produce the LC-MS profiles **(right panels)**. The lower abundant structures found in the brain were separately plotted and further magnified for better visualization. MALDI-MS/MS on two of the major sulfated brain N-glycans at *m/z* 3,212 **(C)** and 2,865 **(D)** afforded characteristic ions (Yu et al., 2009) that support the assignment of the implicated sulfated glycotope as annotated. More detailed structural assignments including the location of sulfate were afforded by nano-LC-MS/MS analysis of the major disulfated N-glycans, the overlaid XICs of which is shown in **(E)** and the representative MS^2^ spectra of doubly charged precursor in **(F)**. Terminal 3-O-sulfated Hex is collectively identified by diagnostic ions at *m/z* 153 and 181 together with the E_1_ and B_1_ ions at *m/z* 253 and 283 (F-i), terminal 4-O-sulfated HexNAc as found in sulfated LacdiNAc by ^3,5^A_1_ and B_1_ ions at *m/z* 167 and *m/z* 324 (F-ii), internal GlcNAc-6-O-sulfate in sulfated NeuAc-Gal-GlcNAc by the diagnostic ions at *m/z* 195, 234, and 264 (F-iii), and the 3-O-sulfated HexA in HNK-1 by ions described in the text (F-iv). A series of singly charged molecular ions from intestine (B) can be additionally assigned as oligomannose structures carrying Man-6-phosphate ± GlcNAc, and be distinguished from isobaric structures in the brain (A) containing instead sulfated HNK-1, based on diagnostic MS^2^ ions (Supplementary Figures S5, S6). Naming and cartoon representation of cleavage ions have been described and fully referenced in our previous work (Yu et al., 2009; Cheng et al., 2015).

Switching to negative mode MS analysis for fractions containing negatively charged permethylated glycans, MALDI-MS analysis detected two major series of signals for the brain sample (Figure 3A). One can be tentatively assigned as oligomannose N-glycans substituted with a Man-6-phosphate (M6P) or M6P-GlcNAc, which were also detected in the intestine sample (Figure 3B). These M6P-containing N-glycans, if present, are often co-fractionated and detected alongside monosulfated N-glycans (Yu et al., 2013), as would be expected from their negatively charged property. The other series of signals can be assigned as sulfated complex-type N-glycans with ±NeuAc-sialylated LacNAc antennae, some of which are further extended by galactosylation. Both NeuAc-Gal-GlcNAc and Gal-(NeuAc)Gal-GlcNAc glycotopes can be sulfated to afford the B_3_ ions at *m/z* 889 and 1,093, respectively, under negative mode MALDI MS/MS (Figure 3C). Interestingly, MALDI-MS/MS on the sulfated N-glycan at *m/z* 2,865 shows that in addition to a sulfated NeuAc-Gal-GlcNAc, the occurrence of another sulfated HexA-Hex-HexNAc glycotope based on a prominent B_3_ ion at *m/z* 746, accompanied by an E_3_ ion at *m/z* 675, ^3,5^A_3_ ion at *m/z* 589, and a D ion at *m/z* 1,140 (Figure 3D, ion types as defined in Yu et al., 2009; 2013).

Due to insufficient sensitivity to detect the low-mass diagnostic ions, the exact location of sulfate cannot yet be determined from MALDI-MS/MS analyses of these monosulfated N-glycans, while their high *m/z* value (>2,500) as singly charged species in negative mode exceed the normal mass range for efficient MS/MS data acquisition by LC-MS/MS. However, the doubly charged disulfated N-glycans (Figure 3E) did afford productive MS/MS to allow additional identification of sulfated glycotopes that can be assigned as sulfated LacNAc, sulfated LacdiNAc, and sulfated sialyl LacNAc, with the sulfate localized to terminal GalNAc, terminal Gal, and internal GlcNAc, respectively, by previously defined diagnostic ions (Cheng et al., 2015; Yu et al., 2018) (Figure 3F). Importantly, a relatively abundant disulfated N-glycan was identified by MS/MS as carrying a sulfated HexA-Hex-HexNAc on both antennae. It afforded not only the same B_3_ and ^3,5^A_3_ ions similarly produced by MALDI-MS/MS at *m/z* 746 and 589, respectively, but also B_2_ and E_2_ ions at *m/z* 501 and 471, and a set of ions at *m/z* 181, 267, 297, and 371 (Figures 3F–3iv), which collectively localize the sulfate to the 3-position of a terminal HexA and unambiguously define a SO_3_-3HexA-3Hex-4HexNAc sequence corresponding to the HNK-1 epitope, SO_3_-3GlcA-3Gal-4GlcNAc.

Unexpectedly, when attempting to further verify the singly charged M6P-containing structures with and without additional Fuc by examining their LC-MS/MS spectra, a similar set of diagnostic ions for the HNK-1 epitope, namely, *m/z* 181, 267, 297, 371, 589, and 746, were commonly produced by all the major peaks at *m/z* < 2,500 (Supplementary Figure S5). In the absence of ions indicative of other sulfated glycotopes, the glycosyl compositions of these structures are consistent with them being hybrid type structures carrying a single HNK-1 antenna and extra Man, with and without core fucosylation, as annotated in Figure 3A. This is in stark contrast with the isobaric structures detected in the intestine sample (Figure 3B), which did carry M6P-GlcNAc and not HNK-1 epitope. In the brain, only the precursors at *m/z* 1839 and 2084 afforded the expected MS^2^ ions for M6P and M6P-HexNAc, respectively (Supplementary Figures S5, S6). The presence of phosphate, instead of sulfate, is not only distinguished by virtue of the phosphate moiety, which can be further O-methylated or substituted by a HexNAc, but also by their accurate masses. For example, the B ion for sulfo-HexA at *m/z* 297.03 (Supplementary Figure S5) differs from that for phospho-Hex carrying an O-methyl group on the phospho moiety at *m/z* 297.07 (Supplementary Figure S6). The unambiguous identification of M6P-containing N-glycans is supportive of previous reports (Fan et al., 2010; Castonguay et al., 2012), suggesting that the lysosomal targeting pathway for enzymes tagged with M6P is conserved in adult zebrafish.

Sulfated N-glycans were not convincingly detected for the ovary samples. For the intestine, a distinctly different set of monosulfated N-glycans were identified (Figure 3B). While the low abundance of detected peaks prevented productive MALDI-MS/MS and hence allowed only composition assignment, the HNK-1–carrying peak detected in the brain is conspicuous by its absence. The tentative assignment based on the corresponding non-sulfated N-glycan structures identified (Figure 1A) suggests that a majority of intestine sulfated N-glycans carry KDN-sialylated or non-sialylated LacNAc, along with a non-extended terminal HexNAc. This is supported by the later elution time of the KDN-sialylated species on the LC time scale, compared to NeuAc/NeuGC-sialylated glycans.

### Sulfated O-Glycans

**FIGURE 4:**
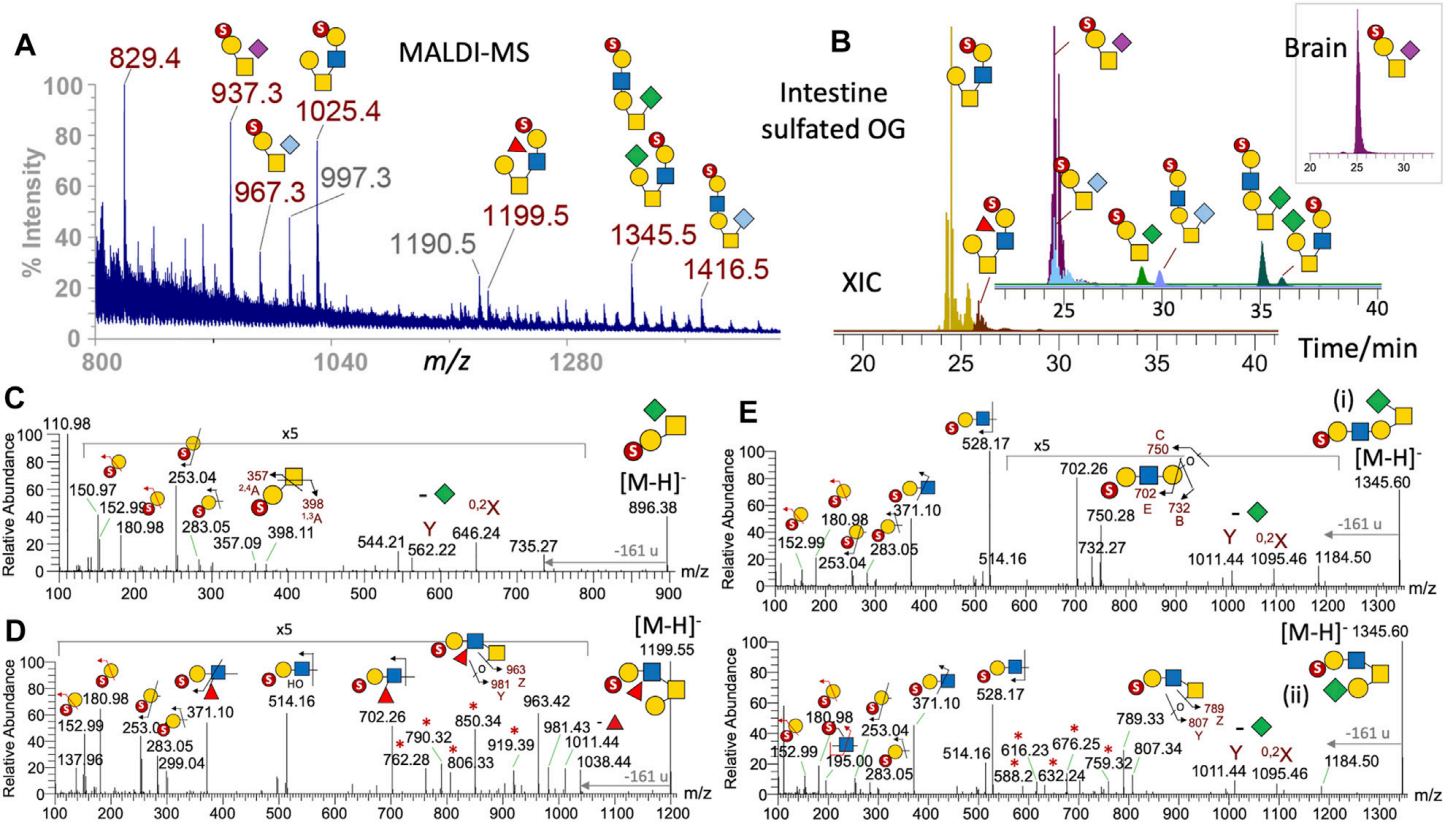
Sulfated O-glycans of adult zebrafish. The sample fractions containing permethylated mono- and multiply sulfated O-glycans derived from the brain, intestine, and ovary were first screened by MALDI-MS, which detected assignable signals only in the brain and intestine monosulfated fractions. The former contained a single [M-H]^-^ signal at *m/z* 937, which was also found among a range of other signals in the intestine sample **(A)**. Full structural assignment was based on further nano-LC-MS/MS analysis, and the XICs of each identified intestine mono-sulfated O-glycans were overlaid to reconstruct a filtered LC-MS profile **(B)**. Two non-sialylated core 2 structures were eluted around the same time as the NeuAc/NeuGc-sialylated core 1 structures, and their overlaid XICs were separately plotted using the same intensity scale and time axis in (B) for better visualization. Similar ion extraction revealed only a sulfated NeuAc-sialylated core 1 structure in the brain (inset at top right). Representative negative mode MS/MS establishing the sialylated core 1 and non-sialylated core 2 structures is shown in **(C)** and **(D)**, respectively. The diagnostic low-mass ions at *m/z* 153 and 181 together with the E_1_ and B_1_ ions at *m/z* 253 and 283 collectively identify a terminal 3-O-sulfated Hex commonly found in all sulfated O-glycans identified here, and the sulfated N-glycans (Figure 3F). The additional ^2,4^A_2_ and ^1,3^A_2_ ions at *m/z* 357 and 398 in (C) indicate that the 3-O-sulfated Hex is 3-linked to an HexNAc, as would be expected for the core 1 structure, whereas the ^3,5^A_2_ ion at *m/z* 371 in (D) implies that it is 4-linked to the HexNAc, as found also in all core 2 structures. The MS^2^ spectra of the sulfated NeuAc/NeuGc-sialylated core 1 structures (not shown) are similar to that sialylated by KDN (C) with the loss of the different sialic acids producing the same Y and ^0,2^X ions at *m/z* 562 and 646. The KDN-sialylated O-glycans at *m/z* 1,345 were clearly resolved into 2 distinct isomeric peaks by LC (B) and their respective core 1 and 2 structures unambiguously established by MS/MS **(E)**. Ion series marked with asterisks are characteristic satellite ions associated with the sulfated fucosylated LacNAc (D) and LacNAc (E) located on the 6-arm of core 2 structures. All diagnostic ions including the loss of 161 u from the GalNAcitol have been described previously (Cheng et al., 2015).

The only prominent sulfated O-glycans detected in the brain by both MALDI and LC-MS/MS is a simple core 1 structure, with 3-O-sulfate on the Gal and NeuAc on GalNAc (Figure 4, inset). The same structure is also a major component in the intestine, but a less amount of NeuGc and KDN-sialylated versions were also (Figures 4A,B), consistent with the overall organ-specific sialylation pattern. Another prominent sulfated O-glycan of the intestine is a simple core 2 structure, with a 3-O-sulfated Gal-GlcNAc on the 6-arm, which can also be fucosylated, or additionally KDN-sialylated on the 3-arm. This KDN-sialylated core 2 structure is readily distinguished by both LC (Figure 4B) and MS/MS (Figures 4E–4ii) from another isomeric extended core 1 structure carrying instead a 3-O-sulfated Gal-GlcNAc on the 3-arm and a KDN on the 6-arm (Figures 4E–4i). In all cases, the 3-O-sulfate on terminal Gal is defined by the aforementioned set of characteristic low-mass ions at *m/z* 153, 181, 253, and 283 (Figures 4C–E), whereas the ^3,5^A_2_ ion at *m/z* 371 indicates that the 3-O-sulfated Gal is 4-linked to GlcNAc. If the sulfated LacNAc is carried on the 6-arm of a core 2 structure, the diagnostic B_2_ ion at *m/z* 528 would be accompanied by a distinct series of satellite ions at *m/z* 588, 616, 632, 676, 745, and 759, and the Z_1_ ion at *m/z* 789 resulting from various modes of cleavages across the GalNAcitol (Figures 4E–4ii). For a sulfated fucosylated LacNAc on the 6-arm, the corresponding B_2_ and its satellite ions would be shifted accordingly by a Fuc increment (174 u) to *m/z* 702, 762, 790, 806, 850, 919, and 963 (Figure 4D). If the sulfated LacNAc is carried on an extended core 1 structure, a triplet of E_3_, B_3_, and C_3_ ions at *m/z* 702, 732, and 750 will be produced, instead of the 6-arm–associated series of ions (Figures 4E–4i). These previously established negative mode fragmentation characteristics of permethylated sulfated O-glycans (Cheng et al., 2015) were reproducibly detected here to allow for an unambiguous assignment of all the major intestine sulfated O-glycan structures.

## Conclusion

Previous glycomic analyses of zebrafish along its developmental line, from oocyte to adult, showed a wide spatiotemporal structural variability on protein- and lipid (glycosphingolipids, GSL)- associated glycans, as well as free glycoprotein–derived oligosaccharides (Takemoto et al., 2005; Guerardel et al., 2006; Moriguchi et al., 2007; Chang et al., 2009; Hanzawa et al., 2017; Yamakawa et al., 2018). Remarkably, a survey of more than 600 glycan molecules in eight organs of adult zebrafish underpinned a yet unexpected level of tissue specificity in vertebrates from both qualitative and quantitative points of view that could be partially correlated with the expression levels of biosynthetic enzymes coding genes (Yamakawa et al., 2018). The structural variability concerns not only the peripheral regions of glycans (Fuc, Sia, extra βGal) but also the internal region (LacNAc, LacDiNAc, branching, elongation, oligomannose vs complex types) and the core (O-glycan core types, GSL families), which suggests an exquisite tissue specificity in regulated glycan biosynthesis. Among the screened organs, the brain, ovary, and intestine revealed unique traits characterized by the expression of very different forms of sialic acids (Neu5Ac, Neu5Gc, and KDN) on specific glycoprotein- and glycolipid-associated glycotopes. In that respect and many others, the combination of MALDI-MS analysis of permethylated sialylated glycans and HPLC analysis of DMB-derived sialic acids used previously only partly accounted for the quantitative variability of glycotopes, particularly the sialylated ones, among various organs. In contrast, the glycotope-centric LC-MS/MS approach used here allowed a more sensitive, more precise, and unbiased quantitative comparison of the nature and level of sialylation by directly assessing the sialic acid species, as well as the sialylated glycotopes expressed on individual glycans (Khoo 2021), confirming that the brain, ovary and intestine preferentially express Neu5Ac, Neu5Gc, and KDN on their glycans, respectively.

Such a strong glycosylation tropism toward distinct sialic acids can only be partially rationalized by the differential expression of cognate sialylation enzymes (Yamakawa et al., 2018). The low level of Neu5Gc synthesis in the brain is substantiated by the relatively low expression of CMP-Neu5Ac hydroxylase (CMAH) compared to other tissues, as reported previously (Malykh et al., 1998). On the contrary, the overwhelming presence of Neu5Gc on ovary glycoconjugates cannot be explained from a functional or biosynthetic point of view, considering in particular the almost exclusive expression of Neu5Ac in the testis (Yamakawa et al., 2018). Finally, the presence of high level of KDN in intestine glycans, as revealed here and previously, may find its origin in a so far unique mechanism of scavenging and metabolic incorporation of bacterial sialic acids from microbiota. Our current glycotope-centric LC-MS/MS approach not only provides an unbiased quantitative assessment of these sialylation differences but also allows resolving close structural isomers otherwise difficult to distinguish by MALDI-MS/MS, as demonstrated here by the identification of a previously unidentified core-3 KDN substituted *O*-glycan in the intestine. Moreover, it successfully led to uncovering of the tissue-specific expression of zebrafish sulfated glycans that were so far overlooked.

The obtained sulfoglycomic data demonstrate that sulfation patterns also vastly differ in the three chosen organs from both qualitative and quantitative aspects. First of all, on the basis of quantitative yield, only the brain and intestine, and not the ovary, were shown to express both sulfated N- and O-linked glycans. Second, the particular sulfated glycans detected in the intestine and brain are very different. Of particular interest, a significant proportion of brain N-glycans were found to bear one or two HNK-1 epitopes. In mammals, HNK-1 is known to be carried by only a few specific proteins in the central nervous system, including neural cell adhesion molecules, L1 and AMPA-type glutamate receptor 2, at the terminal non-reducing end of N-glycans and O-Man glycans (Morise et al., 2014; Morise et al., 2017). The two main enzymes involved in the synthesis of terminal SO_3_-3GlcA epitopes, the glucuronyl transferase GlcAT-P and the sulfotranferase HNK-1ST, have been shown to be expressed in the zebrafish spinal cord, but their product was not identified (Ma et al., 2017). Although to our knowledge, their exact quantity has not been established in the brain of vertebrates, HNK-1–carrying N-glycans are believed to be minor components, as illustrated by the necessity to enrich or purify the carrier glycoproteins from large amounts of brain tissues to observe them (Liedtke et al., 2001). In contrast, the relatively intense MS signals detected among the enriched sulfated N-glycans without further purification strongly suggests that HNK-1–carrying N-glycans are major components in the zebrafish brain, which opens up new routes for the functional analysis of HNK-1 in this model organism.

Along with HNK-1 and LacdiNAc sulfated on the C4 position of GalNAc, which are both brain-specific, a number of other major sulfated glycotopes have been identified on the N-glycans in both the brain and intestine including LacNAc sulfated on the C3 position of Gal and C6-position of GlcNAc, in accordance with the identification of numerous orthologs of Gal-3-O-sulfotransferases and GlcNAc-6-O-sulfotransferases in zebrafish genomes (https://zfin.org). Comparing non-sulfated and sulfated N-glycans profiles shows that most neutral and sialylated N-glycans from the brain and intestine can be further substituted by one or two sulfate groups in the aforementioned positions. However, it is noteworthy that none of the glycans bearing the so-called zebrafish glycotope Galβ1-4 [Sia (α2-3)]Galβ1-4(Fucα1-3)GlcNAc (Guerardel et al., 2006) was found to be sulfated. Again, detailed analysis of the sulfated glycans underpinned the existence of a tightly regulated glycosylation not only at the level of the tissues but also at the level of individual glycans.

## Data Availability

The datasets presented in this study can be found in online repositories. The names of the repository/repositories and accession number(s) can be found below: GlycoPOST (GPST000224.0).
