# Supplementary material for: Discovery Sulfoglycomics and Identification of the Characteristic Fragment Ions for High-Sensitivity Precise Mapping of Adult Zebrafish Brain–Specific Glycotopes
Source: Front Mol Biosci. 2021 Dec 20;8:771447. doi: 10.3389/fmolb.2021.771447 (PMC8721812; doi:10.3389/fmolb.2021.771447)
Supplement: Supplementary file 1 [file DataSheet1.PDF]

Discovery sulfoglycomics and identification of the characteristic fragment ions for high sensitivity precise mapping of adult zebrafish brain-specific glycotopes

Huan-Chuan Tseng[1,2], Cheng-Te Hsiao[1], Nao Yamakawa[3], Yann Guérardel[4,5], Kay-Hooi Khoo[1,2,*]

[1] Institute of Biological Chemistry, Academia Sinica, Taiwan

[2] Institute of Biochemical Sciences, National Taiwan University, Taiwan;

[3] University Lille, CNRS, INSERM, CHU Lille, Institut Pasteur de Lille, US 41-UMS 2014-PLBS, Lille, France.

[4] Université de Lille, CNRS, UMR 8576 – UGSF - Unité de Glycobiologie Structurale et Fonctionnelle, F- 59000 Lille, France.,

[5] Institute for Glyco-core Research (iGCORE), Gifu University, Gifu, Japan.

*Correspondence to: Kay-Hooi Khoo, Email: kkhoo@gate.sinica.edu.tw

---

Supplementary Material

1. Supplemental Figures S1-S3 (this file)

   Fig S1. MALDI-MS profiles of permethylated non-sulfated N-glycans from brain, intestine and ovary.

   Fig S2. MALDI-MS profiles of permethylated non-sulfated O-glycans from brain, intestine and ovary.

   Fig S3. Individual extracted ion chromatogram plots of major non-sulfated N-glycans from brain, intestine and ovary.

   Fig S4. Annotated HCD MS^2 spectra of the identified major non-sulfated N-glycans.

   Fig S5. Annotated negative mode HCD MS^2 spectra of the identified HNK-1 carrying hybrid type N-glycans from brain.

   Fig S6. Annotated negative mode HCD MS^2 spectra of the identified Man-6-phosphate carrying N-glycans from brain and intestine.

2. Supplemental Tables S1-S6 (available separately as Excel file)

   Listing of productive HCD-MS^2 scans acquired on the permethylated non-sulfated N- and O-glycans from brain, intestine and ovary, with precursor ions that can be fitted to reasonable glycosyl compositions.

3. Supplemental Tables S7-S10

   Listing of permethylated sulfated N- and O-glycans identified from brain and intestine, with their expected versus detected *m/z* values and assigned glycosyl compositions.

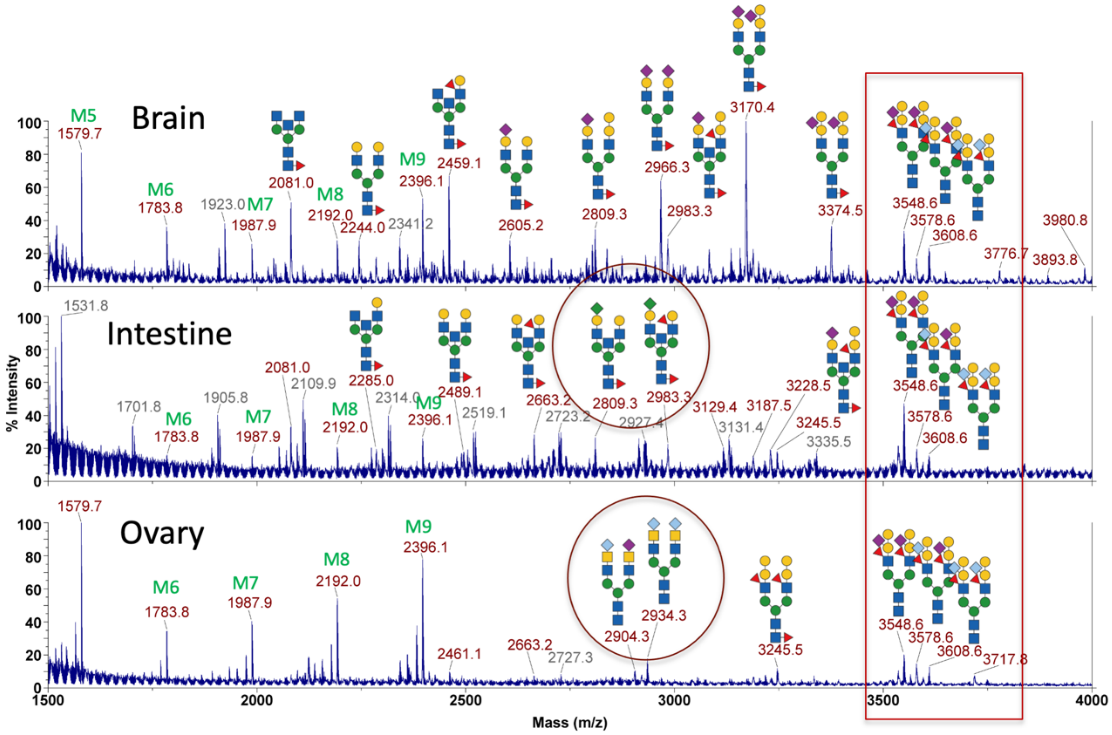

Figure S1. **MALDI-MS profiles of permethylated non-sulfated N-glycans from brain, intestine and ovary**. Major glycans were assigned based on glycosyl composition and subsequent LC-MS/MS data. M5-M9 denote high mannose N-glycans, $Man_5GlcNAc_2$ – $Man_9GlcNAc_2$. The N-glycans circled are unique to intestine and ovary. The N-glycans boxed are common to all three organs.

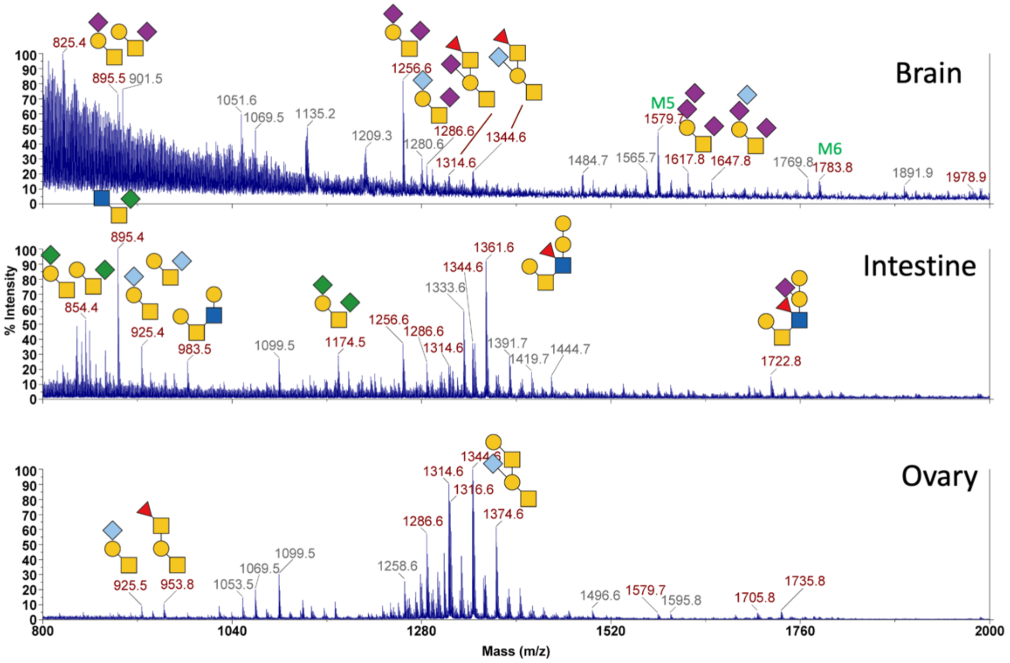

Figure S2. **MALDI-MS profiles of permethylated non-sulfated O-glycans from brain, intestine and ovary.** Major glycans were assigned based on glycosyl composition and subsequent LC-MS/MS data. Unassigned or contaminant peaks are labeled by its *m/z* values in grey.

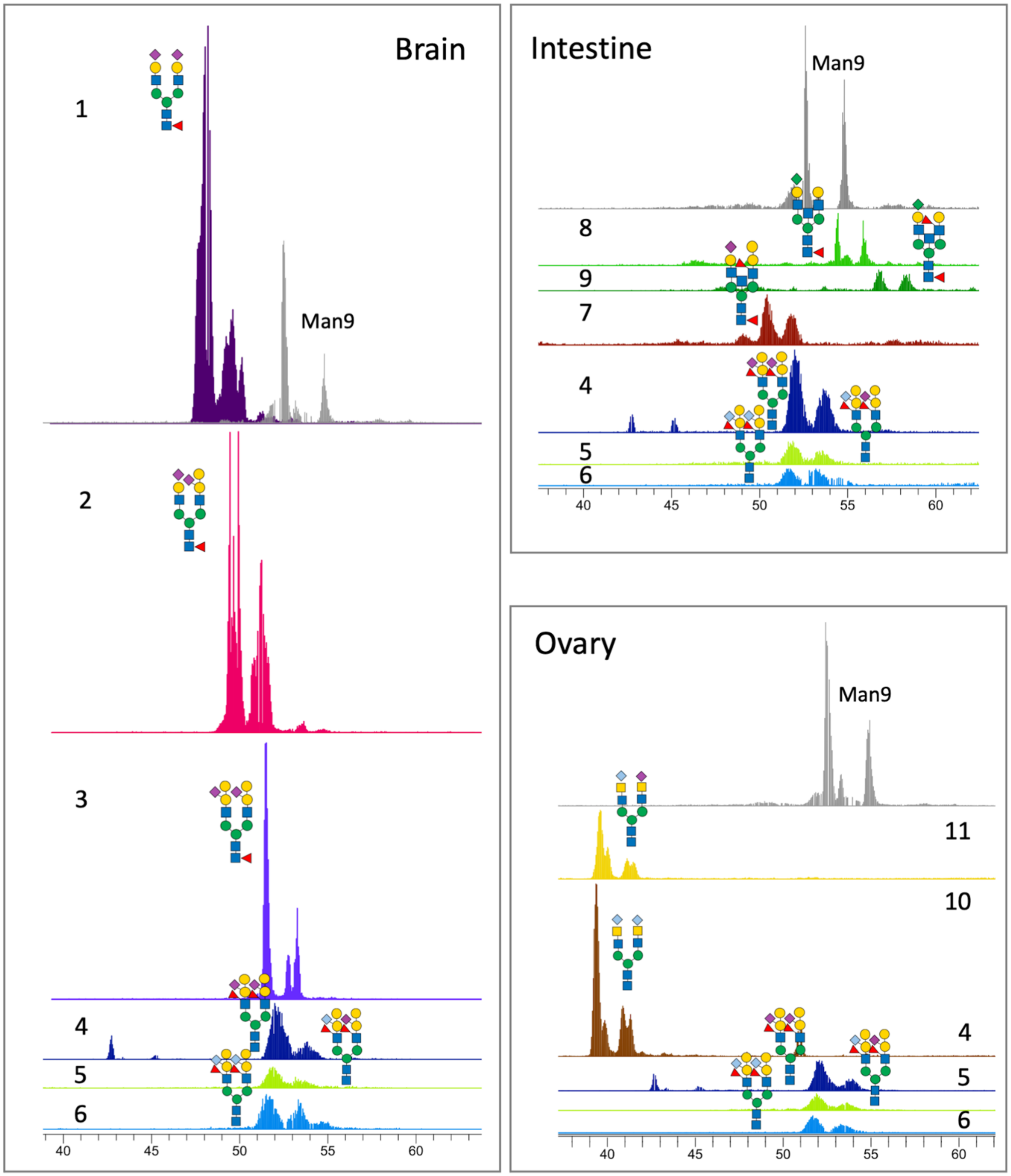

Fig S3. Individual extracted ion chromatogram plots of major non-sulfated N-glycans from brain, intestine and ovary. The same overlaid plots similarly normalized to that of the commonly found $Man_9GlcNAc_2$ (M9) structure in each of the three organs are presented in Fig. 1A and the 11 distinct major complex type structures identified are similarly named 1-11 here. Due to non-reduced reducing end, each was separated into at least 2 peaks by their reducing end anomeric configurations. Additional isomeric structures contributed to extra peaks, which were not individually delineated. Note that structures 4, 5 and 6 are commonly found in all three organs at comparable level relative to M9, with least amount found in ovary. Additional information can be found in the legend to Fig. 1.

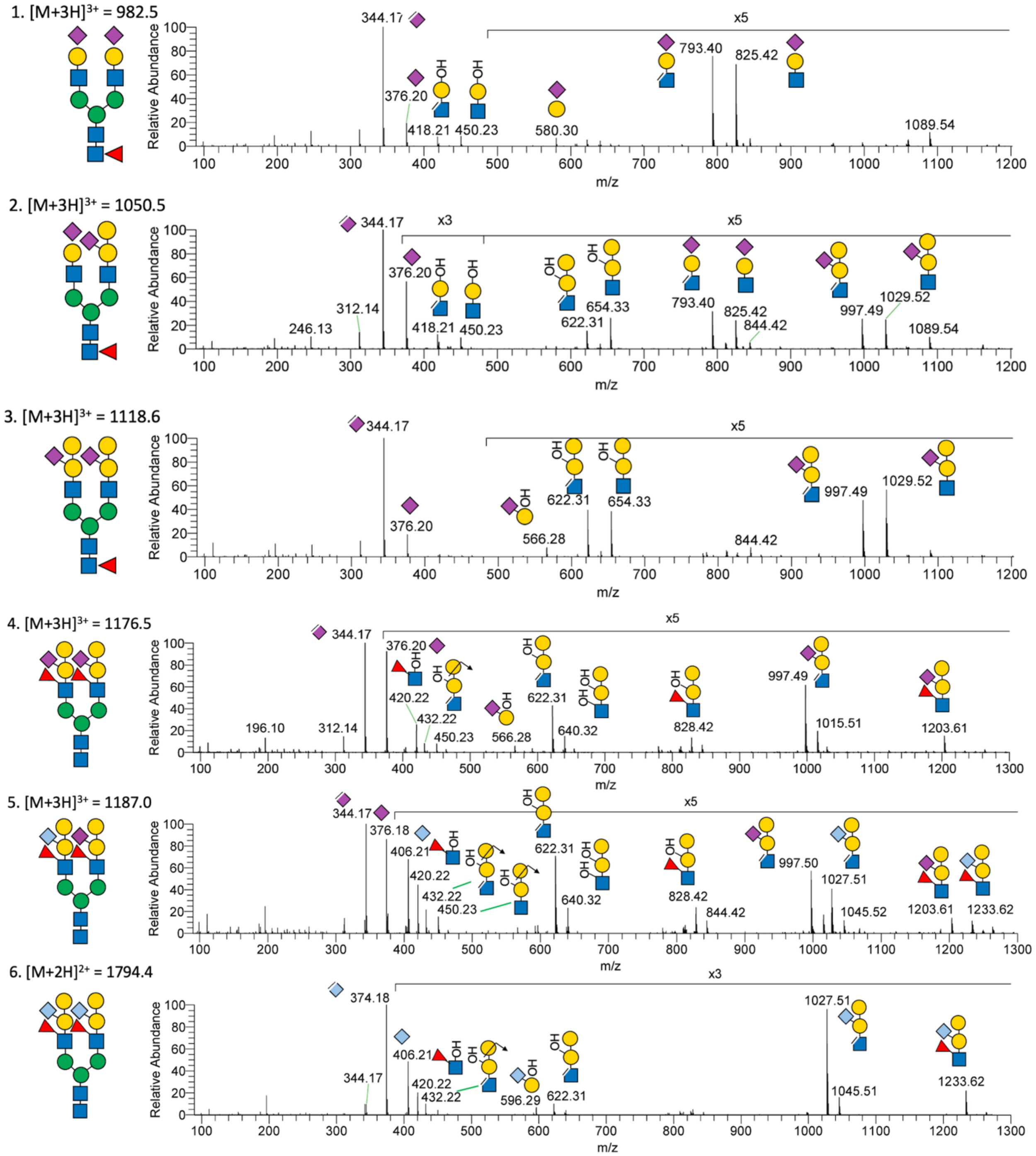

Fig S4. Annotated HCD MS² spectra of the identified major non-sulfated N-glycans. The best spectra for each of the unique N-glycans identified are shown. It should be noted that, in some cases, the MS² ions could be contributed by more than one co-eluting, co-isolated isomeric or isobaric parent structures within the precursor isolation window width. The deduced N-glycan structures 1-11 (shown here and in Fig 1) are simply the best fit structures consistent with most, if not all, of the fragment ions. Continue to next page for structures 7-11.

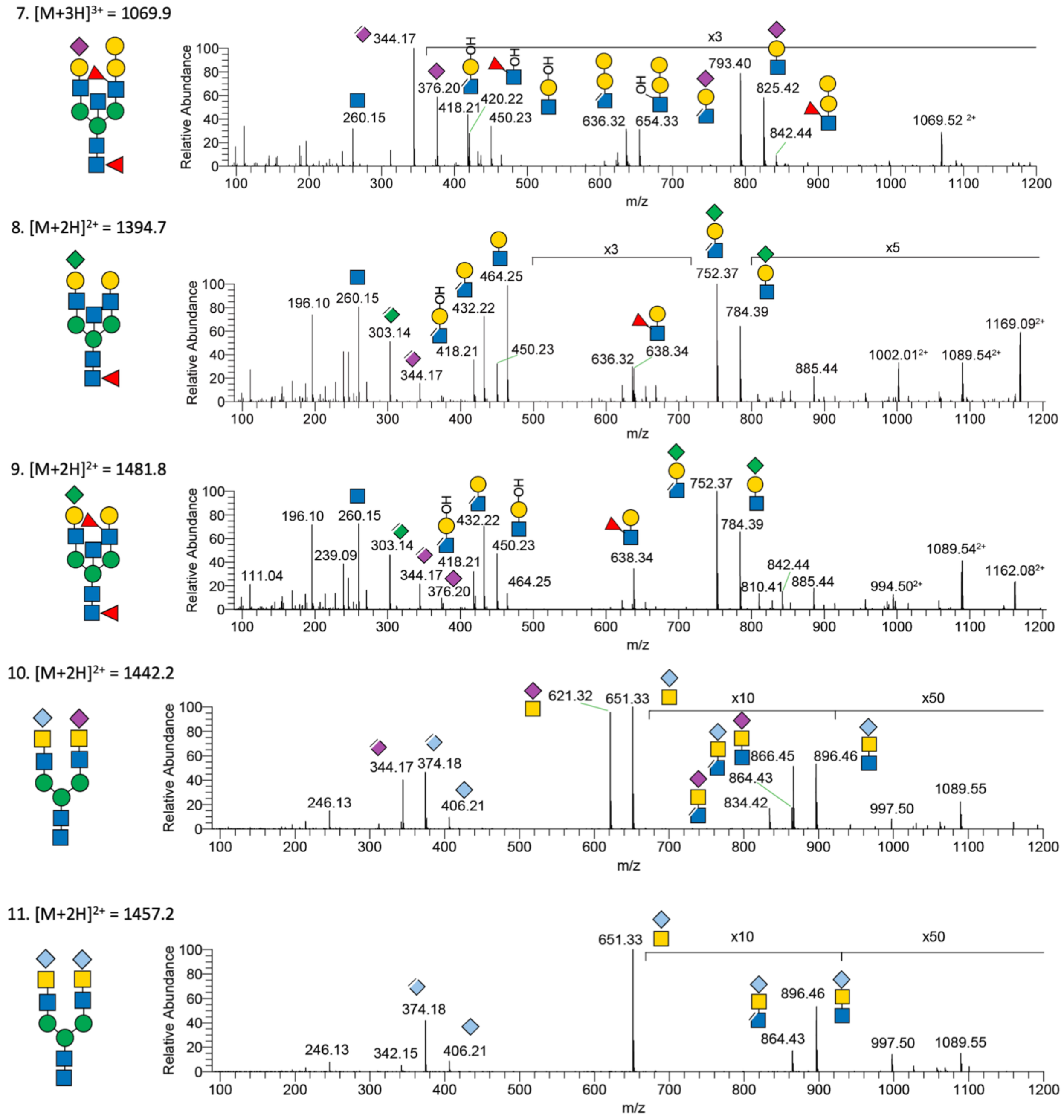

Fig S4. Annotated HCD MS² spectra of the identified major non-sulfated N-glycans. Continued from previous page, showing here the MS² spectra for structures 7-11.

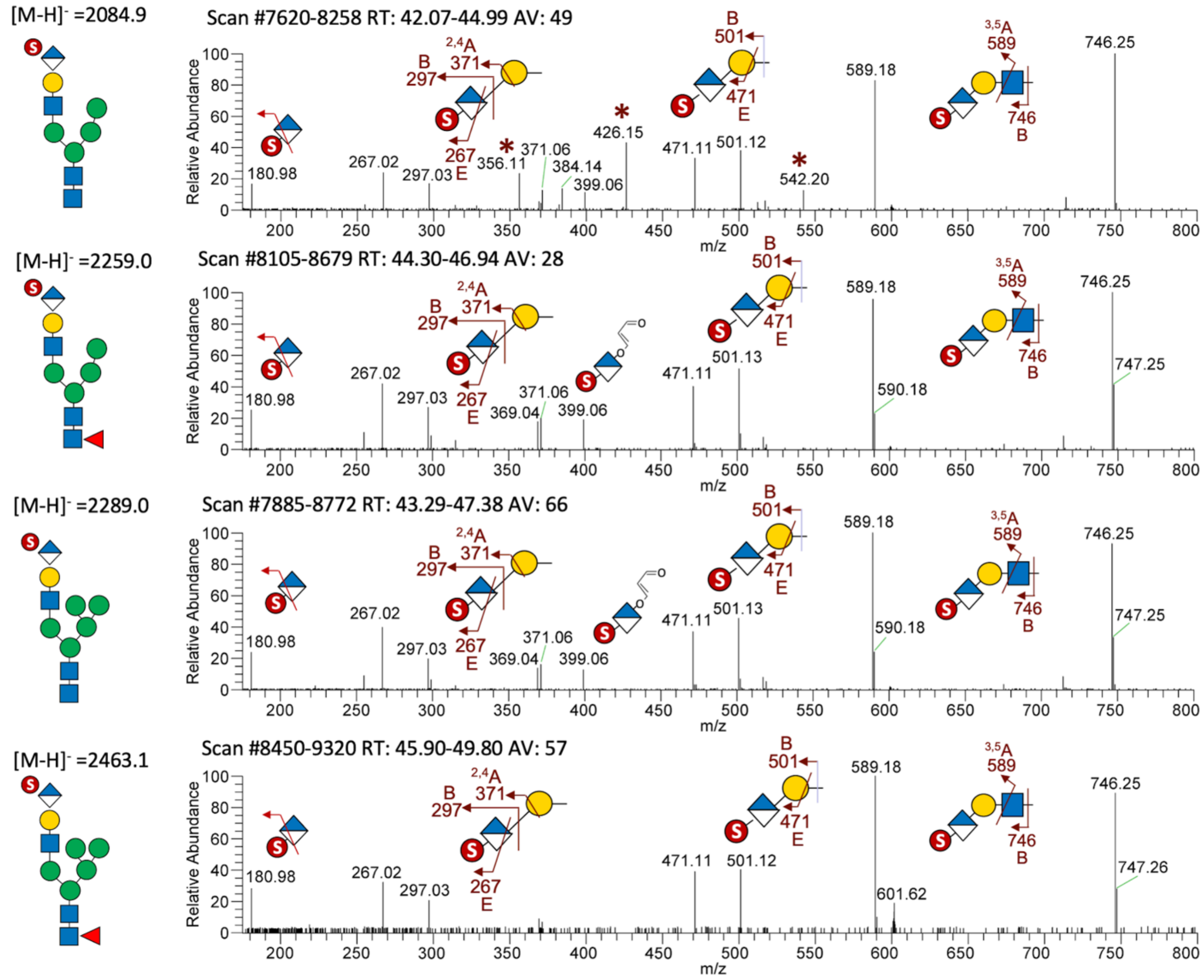

Fig S5. Annotated negative mode HCD MS² spectra of the identified HNK-1 carrying hybrid type N-glycans from brain. LC-MS² spectra were acquired on the singly charged [M-H]- precursors, with their *m/z* values and deduced structures shown in the left panel. Only the low mass region (m/z <800) contained useful signals including the few diagnostic ions of the 3-O-sulfated HNK-1 glycotope, as annotated. The MS² spectrum for the doubly charged complex type N-glycans carrying 2 HNK-1 glycotopes is shown in Fig. 3F(iv). The hybrid type structure with 1 or 2 Man on the other arm of the trimannosyl core and the core fucosylation were deduced based on absence of other terminal sulfated glycotopes and any extra HexNAc. Note that the MS² ions produced by the precursors at *m/z* 2084 and 2289 from brain as shown here are very different from those produced by the isobaric Man-6-phosphate-GlcNAc containing precursors of similar *m/z* values from intestine (shown in Fig. S6). However, the precursor at *m/z* 2084 from brain also afforded diagnostic ions of Man-6-phosphate-GlcNAc (*), indicative of its presence alongside the HNK-1 carrying isobaric structure.

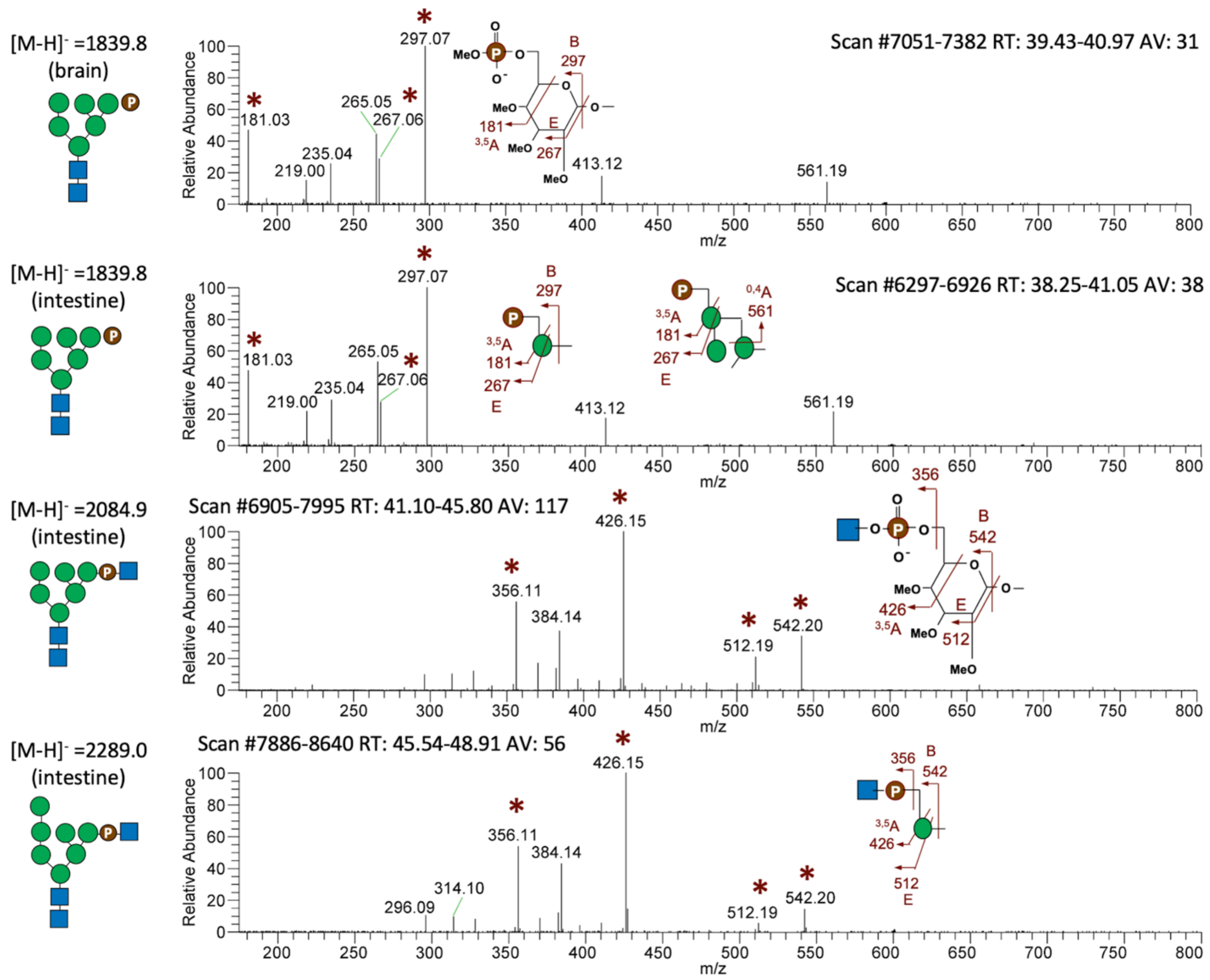

Fig S6. Annotated negative mode HCD MS² spectra of the identified Man-6-phosphate carrying N-glycans from brain and intestine. LC-MS² spectra were acquired on the singly charged [M-H]⁻ precursors, with their *m/z* values and deduced structures shown in the left panel. Only the low mass region (m/z <800) contained useful signals including the few diagnostic ions (*) of the Man-6-phosphate and Man-6-phosphate-GlcNAc, as reported previously (Yu et al, 2013). The Man-6-phosphate moiety could be carried on a terminal or penultimate Man (Manα1-2Man-), whereas the Man-6-phosphate-GlcNAc detected here appears to be carried only on the terminal Man. The exact oligomannose arrangement could not be defined by the afforded MS² and the cartoon structures drawn here represent only one of the possibilities. Note that the MS² ions produced by the precursors at *m/z* 2084 and 2289 from intestine as shown here are very different from those produced by the isobaric precursors of similar *m/z* values from brain (shown in Fig. S5). For the diagnostic ions, the phosphate carrying ions at *m/z* 181.03, 267.06 and 297.07 can be distinguished from those derived from 3-O-sulfated HexA at *m/z* 180.98, 267.02 and 297.03, consistent with their expected accurate masses.

Ref: Yu, S.Y., Chang, L.Y., Cheng, C.W., Chou, C.C., Fukuda, M.N. and Khoo, K.H. (2013) Priming mass spectrometry-based sulfoglycomic mapping for identification of terminal sulfated lacdiNAc glycotope. *Glycoconj J*. 30, 183-194. doi:10.1007/s10719-012-9396-z

Table S7. LC-MS data for assigned adult zebrafish brain monosulfated N-glycans.

RT	Monoisotopic m/z for [M-H]-			Intensity (XIC, LC-MS)	Z	Assigned Glycosyl Composition + Sulfate/Phosphate							
	Theorectical	MALDI MS	LC-MS			dHex	Hex	HexNAc	NeuAc	NeuGc	GlcA	Sulfate	Phosphate
40.13	1839.8426	1839.7241	1839.8521	105872673	-1	0	6	2	0	0	0	0	1
42.17	2084.9326	2084.8076	2084.9309	88534741	-1	0	5	3	0	0	1	1	0
44.49	2259.0218	2258.8562	2259.0127	477102458	-1	1	5	3	0	0	1	1	0
44.49	2289.0324	2288.8662	2289.0217	540221756	-1	0	6	3	0	0	1	1	0
46.77	2463.1216	2462.9346	2463.1135	144236041	-1	1	6	3	0	0	1	1	0
45.81	2647.2417	2647.0168	2647.2354	37108766	-1	1	5	4	1	0	0	1	0
49.36	2865.3218	2865.0823	2865.3113	33549925	-1	1	5	4	1	0	1	1	0
49.76	3008.4154	3008.1533	3008.4070	62337070	-1	1	5	4	2	0	0	1	0
51.69	3212.5162	3212.2246	3212.4978	66815243	-1	1	6	4	2	0	0	1	0
53.60	3416.6159	3416.3044	3416.6008	7446029	-1	1	7	4	2	0	0	1	0

Table S8. LC-MS data for assigned adult zebrafish intestine monosulfated N-glycans.

RT	Monoisotopic m/z for [M-H]-			Intensity (XIC LC-MS)	Z	Assigned Glycosyl Composition + Sulfate/Phosphate							
	Theorectical	MALDI MS	LC MS			dHex	Hex	HexNAc	NeuAc	NeuGc	KDN	Sulfate	Phosphate
38.43	1839.8426	1839.7181	1839.8479	121745580	-1	0	6	2	0	0	0	0	1
42.97	2084.9689	2084.8230	2084.9783	646029806	-1	0	6	3	0	0	0	0	1
45.87	2289.0687	2288.8997	2289.0745	180907290	-1	0	7	3	0	0	0	0	1
42.26	2327.0956	2326.8850	2327.0898	59661814	-1	1	4	5	0	0	0	1	0
39.52	2357.1062	2356.8940	2357.1042	24523984	-1	0	5	5	0	0	0	1	0
43.84	2531.1954	2530.9646	2531.1899	56651108	-1	1	5	5	0	0	0	1	0
51.20	2677.2533	2677.0005	2677.2456	21974525	-1	0	5	5	0	0	1	1	0
52.86	2851.3425	2851.0693	2851.3291	152171693	-1	1	5	5	0	0	1	1	0
53.92	3025.4317	3025.1370	3025.4197	15917331	-1	2	5	5	0	0	1	1	0

Table S9. LC-MS data for assigned adult zebrafish brain disulfated N-glycans.

RT	Theorectical monoisotopic m/z		Observed m/z by LC-MS	Intensity (XIC LC-MS)	Z	Assigned Glycosyl Composition + Sulfate						
	[M-H]-	[M-2H]2-				dHex	Hex	HexNAc	NeuAc	NeuGc	GlcA	Sulfate
31.29	2409.0661	1224.5437	1224.5452	29753726	-2	1	3	6	0	0	0	2
33.66	2613.1659	1306.0790	1306.083	11329525	-2	1	5	5	0	0	0	2
35.04	2729.2153	1364.1040	1364.1052	15842492	-2	1	5	4	1	0	0	2
39.90	2804.1997	1401.5962	1401.5967	20469764	-2	1	5	4	0	0	2	2
37.37	2947.2943	1473.1435	1473.1456	12324940	-2	1	5	4	1	0	1	2
37.42	3090.3889	1544.6908	1544.6927	26484022	-2	1	5	4	2	0	0	2
42.50	3294.4887	1646.7407	1646.7404	14829109	-2	1	6	4	2	0	0	2
46.55	3498.5864	1748.7906	1748.7921	6006253	-2	1	7	4	2	0	0	2

Table S10 LC-MS data for assigned adult zebrafish monosulfated O-glycans.

RT	Monoisotopic m/z for [M-H]-			Intensity (XIC, LCMS)	Z	Assigned Glycosyl Composition + Sulfate							Source
	Theorectical	MALDI MS	LC-MS			dHex	Hex	HexNAc	NeuAc	NeuGc	KDN	Sulfate	
25.07	937.4067	937.4319	937.4066	38853547	-1	0	1	1	1	0	0	1	Brain
28.96	896.3802	896.3118	896.3859	90235197	-1	0	1	1	0	0	1	1	Intestine
24.51	937.4067	937.3429	937.4173	491478704	-1	0	1	1	1	0	0	1	Intestine
24.51	967.4173	967.3447	967.4214	188703422	-1	0	1	1	0	1	0	1	Intestine
24.51	1025.4592	1025.3904	1025.4652	603309492	-1	0	2	2	0	0	0	1	Intestine
25.91	1199.5485	1199.4576	1199.5525	109922784	-1	1	2	2	0	0	0	1	Intestine
35.07	1345.6064	1345.5127	1345.6195	191482509	-1	0	2	2	0	0	1	1	Intestine
29.88	1416.6435	1416.5386	1416.6487	72829438	-1	0	2	2	0	1	0	1	Intestine

Note: All m/z values including the expected theorectical masses and experiemtnally detected masses refer to the monoisotopic masses. Intensity of each detected component is based on the peak areas of their respective extracted ion chromatograms (XIC).
